# Recurrence of Herpetic Keratitis after COVID-19 Vaccination: A Report of Two Cases

**DOI:** 10.1155/2022/7094893

**Published:** 2022-05-19

**Authors:** Ali Mahdavi Fard, Jeffrey Desilets, Sangita Patel

**Affiliations:** ^1^Ross Eye Institute, Department of Ophthalmology, Jacobs School of Medicine and Biomedical Sciences, State University at Buffalo, Buffalo, New York, USA; ^2^Research and Ophthalmology Services, Veterans Administration of Western New York Healthcare System, Buffalo, NY, USA

## Abstract

**Background:**

Recurrence of herpetic keratitis following vaccination has been documented following vaccination with the Zostavax, trivalent flu, hepatitis A, and rabies vaccines. The USFDA and WHO have acknowledged that the novel COVID-19 vaccines similarly have a risk of reactive immunologic-based inflammation, namely, myositis, pericarditis, and Guillain-Barré syndrome. *Case Presentation*. We present two patients with latent herpetic keratitis who experienced reactivation of keratitis within weeks of COVID-19 vaccination despite prolonged periods of prior latency. A 52-year-old healthy male with no herpes simplex virus (HSV) keratitis recurrences in two years developed visual decline and patchy stromal haze within 24-48 hours of receiving the second Pfizer-BioNTech (COVID-19 BNT162b2) vaccine. A 67-year-old female with chronic neurotrophic keratitis developed her most severe exacerbation of herpes zoster keratitis in over 10 years occurring 2-3 weeks after her first Moderna (mRNA-1273) vaccine, which was later complicated by bacterial superinfection.

**Conclusions:**

The COVID-19 vaccines work by generating both adaptive humoral and cellular immune responses in humans, including elevation of anti-spike neutralizing antibody titers, antigen-specific CD4+ and CD8+ T-cell responses, and increased levels of proinflammatory cytokines such as interferon gamma (IFN*γ*). The general activation of the T-cell-mediated immune response and proinflammatory cytokines such as IFN*γ* may underlie the role of the COVID vaccines in reactivation of herpetic stromal keratitis and the clinical findings in our reported cases.

## 1. Introduction

Following the historic rollout of the COVID-19 vaccines to the public in early 2021, the United States Food and Drug Administration and World Health Organization have acknowledged a low risk of certain forms of reactive immunologic-based inflammation, namely, myositis, pericarditis, and Guillain-Barré syndrome [1]. We present two patients with chronic herpetic keratitis and prolonged periods of prior latency who experienced reactivation of keratitis within weeks of vaccination.

## 2. Case 1

**Figure 1:**
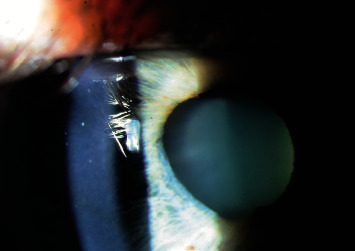
Slit lamp photograph of the right eye of the patient in case 1 depicting corneal stromal haziness and sectoral iris atrophy.

A 52-year-old healthy male had a longstanding history of herpetic keratitis of the right eye. The herpes simplex virus (HSV) stromal keratitis first occurred in 1992, and the patient experienced 3-4 episodes of reactivation per year in the right eye until 2010 after which the frequency decreased to one episode of reactivation every other year with the last episode in 2019. A typical episode would include pruritus, irritation, redness, and photophobia without vision changes and would last 2-3 weeks. These recurrences were treated with topical prednisolone acetate 1%, topical trifluridine, and oral antiviral (acyclovir or valacyclovir). Between episodes, he was maintained on oral antiviral prophylaxis and topical prednisolone acetate 1% 2-3×/week. The topical steroid was discontinued in 2017. The patient received the Zostavax varicella zoster vaccine in March 2020 without incident. He received the Pfizer-BioNTech COVID-19 vaccines (COVID-19 BNT162b2) in March 2021 and April 2021. Within 24-48 hours of receiving the second vaccination, the patient began to experience blurry vision in the right eye which he had not experienced during prior episodes of recurrence of herpetic keratitis. There was no associated pruritus, redness, irritation, or photophobia like his prior episodes. The patient had an eye exam two weeks after the initial symptoms and was found to have 20/40 visual acuity in the right eye with patchy stromal haze and confluent punctate epithelial erosions along the inferior cornea. The patient's prophylactic dose of acyclovir 800 mg daily was increased to 400 mg 5×/day, and topical trifluridine was prescribed at 5×/day in the right eye. One week later, the patient presented to our clinic and vision had declined to 20/60 with new nummular opacities at multiple stromal levels. Sector iris stromal atrophy was noted (Figure 1). Prednisolone acetate 1% drops 5×/day were started with improved vision within 2-3 days. The prednisolone acetate 1% drops were tapered gradually to fluorometholone 0.1%, and antiviral was switched to valacyclovir 500 mg once daily with maintained quiescence of the herpetic keratitis. This patient continues to be followed, and vision in the right eye has stabilized at 20/30.

## 3. Case 2

A 67-year-old female had a history of hypercholesterolemia, hypothyroidism, Hodgkin's lymphoma, and complicated herpes zoster ophthalmicus (HZO) of her left eye. She developed herpes zoster ophthalmicus in 1981 while completing chemotherapy for Hodgkin's lymphoma. At that time, she was hospitalized for 3 months with severe Guillain-Barré syndrome with full-body flaccid paralysis and respiratory compromise without intubation. She had severe ocular involvement which could not be fully evaluated due to her systemic status, and a left eye tarsorrhaphy was maintained through her hospitalization. Despite a severe initial course, with prophylactic bandage contact lens wear for neurotrophic corneal status and fluorometholone 0.1% drops once daily, the patient had maintained 20/40 visual acuity with a clear cornea for over 10 years prior to the current case presentation. The patient received the Zostavax shingles vaccine in 2017 and the Shingrix vaccine in 2019 and 2020 without ocular issues. She believed she experienced COVID-19 infection in March 2020 due to an illness with fever and cough and subsequent positive at-home COVID-19 antibody test but had no ocular issues at that time. The patient received the Moderna COVID-19 vaccines (mRNA-1273) in January 2021 and February 2021. She began to experience blurry vision, redness, and abnormal sensation in her left eye at the end of January 2021, just prior to her second COVID-19 vaccination. Her visual acuity in the left eye had decreased to 20/150, and the corneal exam revealed a 1 × 0.5 mm corneal epithelial defect without infiltrate suggestive of herpetic epithelial keratitis. A bandage contact lens, oral valacyclovir 1 g twice daily, and ofloxacin 0.3% drops 4×/day were initiated with continuation of her baseline fluorometholone 0.1% once daily. A swab of the corneal lesion was submitted for evaluation for herpes simplex virus (1 and 2) and varicella zoster virus by qualitative polymerase chain reaction. The test results were inconclusive due to a low number of cells detected in the specimen by the endogenous control. A stromal infiltrate vs. herpetic stromal keratitis was present the subsequent week at which time bacterial corneal cultures were obtained. Cultures grew *Mycobacterium chelonae*, and amikacin drops 15 mg/mL with oral azithromycin 500 mg weekly for 5 weeks were started. While the ulcer resolved, the healing course was complicated by progressive stromal thinning, iritis, endotheliitis, and difficulty with corneal epithelial healing.

## 4. Discussion

There are multiple known triggers for reactivation of herpetic keratitis. Factors noted for reactivation of HSV keratitis include pyrexia, wind and sun exposure, trauma, and stress [2]. Recurrence of herpetic keratitis following vaccination has been reported following Zostavax vaccination [3]. In addition, reactivation of herpes zoster has been reported after the trivalent flu, hepatitis A, and rabies vaccines [4]. To the list of potential triggers for recurrent herpetic keratitis, we add the COVID-19 BNT162b2 and mRNA-1273 mRNA vaccines.

Recurrent herpetic stromal keratitis is typically the result of cell-mediated immunity against viral DNA antigens in the cornea, rather than active viral replication. Several studies have shown that during recurrent herpetic stromal keratitis, the T-cell profile is different from primary infection [5]. While cytotoxic T-cells are involved in primary disease [6], recurrent disease is most often associated with strong delayed-type hypersensitivity (DTH) responses [7]. The DTH response in recurrent herpetic stromal keratitis occurs as a result of developing an immune response against HSV-1 including antigen-specific CD4+ T-cells of both Th1 and Th2 subsets as well as HSV-1-specific CD8+ T-cells. Similar mechanisms of cell-mediated immunity against viral antigens in the cornea are responsible for the later manifestations of HZO [8]. In HSV stromal keratitis, the CD4+ T-cell production of inflammatory cytokines such as interferon gamma (IFN*γ*) also triggers pathogenic neutrophil infiltration [9].

Since the emergence of the COVID-19 pandemic, two mRNA-based vaccines, BNT162b2 and mRNA-1273, have been introduced that demonstrated a high efficacy rate with an acceptable safety profile for public administration against the SARS-CoV-2 virus. These vaccines work by generating both adaptive humoral and cellular immune responses in humans, including elevation of anti-spike neutralizing antibody titers, antigen-specific CD4+ and CD8+ T-cell responses, and increased levels of proinflammatory cytokines such as interferon gamma (IFN*γ*) [10]. While it is possible that the timing was a coincidence, the general activation of the T-cell-mediated immune response and proinflammatory cytokines such as IFN*γ* may underlie the role of the COVID vaccines in reactivation of herpetic keratitis and the clinical findings in our reported cases.

The COVID-19 vaccines have over 90% efficacy in preventing a disease that was responsible for about 2.6 million deaths worldwide during its first year alone; this medical advancement provides paramount protection to both the individual and society at large. For patients with HSV and VZV with history of ocular involvement, clinicians may wish to observe for ocular changes presenting around the time of vaccination. Patients may also be advised to self-monitor vision and ocular symptoms in the weeks following vaccination and to report any changes immediately.

## Data Availability

All data used in this manuscript are included in the case report.

## References

[1] WHO (2021). Covid-19 vaccines. *Safety Surveillance Manual second edition*.

[2] Al-Dujaili L. J., Clerkin P. P., Clement C. (2011). Ocular herpes simplex virus: how are latency, reactivation, recurrent disease and therapy interrelated?. *Future Microbiology*.

[3] Hwang C. W., Steigleman W. A., Saucedo-Sanchez E., Tuli S. S. (2013). Reactivation of herpes zoster keratitis in an adult after varicella zoster vaccination. *Cornea*.

[4] Walter R., Hartmann K., Fleisch F., Reinhart W. H., Kuhn M. (1999). Reactivation of herpesvirus infections after vaccinations?. *Lancet*.

[5] Keadle T. L., Morris J. L., Pepose J. S., Stuart P. M. (2002). CD4^+^ and CD8^+^ cells are key participants in the development of recurrent herpetic stromal keratitis in mice. *Microbial Pathogenesis*.

[6] Chang E., Galle L., Maggs D., Estes D. M., Mitchell W. J. (2000). Pathogenesis of herpes simplex virus type 1-induced corneal inflammation in perforin-deficient mice. *Journal of Virology*.

[7] Keadle T. L., Morrison L. A., Morris J. L., Pepose J. S., Stuart P. M. (2002). Therapeutic immunization with a virion host shutoff-defective, replication-incompetent herpes simplex virus type 1 strain limits recurrent herpetic ocular infection. *Journal of Virology*.

[8] Baratz K. H. (2012). The role of antiviral therapy after the resolution of acute herpes simplex keratitis or acute herpes zoster ophthalmicus. *Archives of Ophthalmology*.

[9] Lobo A. M., Agelidis A. M., Shukla D. (2019). Pathogenesis of herpes simplex keratitis: the host cell response and ocular surface sequelae to infection and inflammation. *The Ocular Surface*.

[10] Polack F. P., Thomas S. J., Kitchin N. (2020). Safety and efficacy of the BNT162b2 mRNA Covid-19 vaccine. *New England Journal of Medicine*.

